# Caregiving burden, depression, and anxiety among family caregivers of patients with cancer: An investigation of patient and caregiver factors

**DOI:** 10.3389/fpsyg.2023.1059605

**Published:** 2023-03-28

**Authors:** Zhaleh Karimi Moghaddam, Mina Rostami, Alireza Zeraatchi, Jahangir Mohammadi Bytamar, Omid Saed, Saeedeh Zenozian

**Affiliations:** ^1^Department of Radiation Oncology, Vali-e-Asr Hospital, School of Medicine, Zanjan University of Medical Sciences, Zanjan, Iran; ^2^Social Determinants of Health Research Center, Zanjan University of Medical Sciences, Zanjan, Iran; ^3^Department of Emergency Medicine, Valiasr-e-Asr Hospital, Ayatollah Mousavi Hospital, School of Medicine, Zanjan University of Medical Sciences, Zanjan, Iran; ^4^Department of Clinical Psychology, Beheshti Hospital, Zanjan University of Medical Sciences, Zanjan, Iran

**Keywords:** burden, anxiety, depression, family caregiver, cancer patient

## Abstract

**Background:**

Caring for patients with cancer can result in significant burden, anxiety, and depression among family caregivers, leading to alterations in their mental and physical wellbeing. Evidence on the level of cancer caregivers' burden, depression, anxiety, their role in assisting their patients, and other patient and caregiver factors that play in improving/worsening the outcomes, is limited. This study explored the prevalence of caregiving burden, depression, and anxiety with a focus on the patient and caregiver-related factors among cancer family caregivers.

**Methods:**

A cross-sectional study was conducted on the population of caregivers of adult patients with cancer in Zanjan, Iran between 2019 and 2020. The Beck Depression Inventory (BDI), the Beck Anxiety Inventory (BAI), and the Zarit Burden Inventory (ZBI) were used to measure outcome variables. Clinical and basic characteristics of the caregivers and patients were also collected. An independent samples *t*-test, analysis of variance, Pearson's correlation coefficient, and stepwise linear regression were performed using SPSS software version 26.

**Results:**

Mean ± standard deviation age of the caregivers (167 men and 133 women) was 40.77 ± 12.56. Of the caregivers, 46.3, 53, and 30.7% showed severe depression, anxiety, and burden, respectively. There was a significant positive correlation between ZBI with both BDI [*r*_(298)_ = 0.19, *p* < 0.01] and BAI [*r*_(298)_ = 0.20, *p* < 0.01]. Caregiving ≥24 months (*B* = 14.36, *p* < 0.001), outpatient care setting (*B* = −12.90, *p* < 0.001), being retired (*B* = −12.90, *p* < 0.001), depression (*B* = 0.28, *p* < 0.001), supplemental health insurance (*B* = −7.79, *p* < 0.001), being illiterate (*B* = 7.77, *p* < 0.01), surgery (*B* = 8.55, *p* < 0.01), ECOG1 (*B* = 4.88, *p* < 0.01), and patient's age (*B* = 0.11, *p* < 0.05) were found to be significant predictors of caregiving burden.

**Conclusion:**

High levels of depression, anxiety, and burden were observed among the caregivers of patients with cancer. These findings underline the importance of paying close attention to the needs and psychological challenges of this population.

## Background

Chronic and non-communicable diseases are the most important challenges of the current century, leading to increased costs and adverse social effects on patients and communities worldwide (Karbaschi et al., 2015). Cancer is one of the fastest-growing health issues in Iran and all across the globe, and is the second most common disease and the third leading cause of death following cardiovascular disease and accidents (Karbaschi et al., 2015). According to recent statistics, each year, more than 50 million cancer deaths occur throughout the world, and more importantly, 80% of these deaths occur in low- and middle-income countries (Akpan-Idiok et al., 2020).

Due to the increasing incidence of new cancer cases in recent years, the number of people undertaking cancer caregiver roles has been on the rise dramatically (Onyeneho and Ilesanmi, 2021). A remarkable proportion of caregivers are the family members of patients with cancer, who play a noteworthy role in assisting their patients to confront the harsh reality of cancer diagnosis and equip them with both practical and emotional support (Onyeneho and Ilesanmi, 2021). Following the diagnosis of cancer and initiating the treatment process, the patient's family members feel responsible for taking care of their patient. Being in the role of a family caregiver is not usually predictable and optional for the family members and somehow seems inevitable (Abbasi et al., 2013). Indeed, being the main source of support, family caregivers are supposed to play a considerable role in caring for their patients with cancer. The fact that one of their loved ones has to struggle with a terminal disease often disrupts the family routine and makes the family keep a balance between the demands of cancer trajectory and their routines (Coppetti et al., 2019). Furthermore, in light of the fact that the need for caring often arises suddenly and caregivers do not have sufficient prior guidance and preparation, physical and psychological changes may occur (Coppetti et al., 2019).

How undertaking caregiving responsibility may impact the psychological health of caregivers and what its psychological outcomes have always been key questions to be addressed concerning the mental health of caregivers. Stress, anxiety, worry, depression, isolation, and anger are among the psychological outcomes of caregiving that have been examined in the existing literature (Given et al., 2012). Depression and anxiety are among the most frequent psychological consequences reported in previous studies, ranging from 52 to 94% among family caregivers (Thrush and Hyder, 2014). The level of such outcomes might be even higher among caregivers than patients themselves. For instance, it has been confirmed in a population of head and neck cancer and hematological cancer caregivers that they would even develop greater psychological distress than the general population and their patients (Caruso et al., 2017; Kassir et al., 2021). Existing evidence has pointed to the increased levels of stress and psychosocial distress among family caregivers when they have to maintain a balance between their professional careers and domestic duties (Gupta et al., 2022).

Moreover, assuming the role of caregiver imposes a great burden on caregivers, affecting different aspects of their life, including mental health, physical health, and financial status (Given et al., 2012). The fact that cancer care and treatment are costly adds to the financial challenges of caregivers.For example, among patients who are insured against medical costs, the out-of-pocket expenses including deductibles, co-payments, and co-insurance may be enormous under some of the health insurance plans. In addition, there are copayments required to be paid for different services involved in cancer care treatment such as healthcare and medications, nutritional supplements, and meals at the hospital, which can double burden the financial issues (Xiang et al., 2022). In some cases, the unemployment of the patient and the caregiver aggravates these problems (Given et al., 2012). More importantly, the lack of social support and the unnecessary stringent measures of insurance companies in releasing the payments for medical expenses might make the burden even greater (Cejalvo et al., 2021).

It has already been indicated that in caregivers of patients with cancer, there has been a significant positive correlation between caregiver burden and family distress index. High scores of family distress index, along with other factors such as patient gender and time since cancer diagnosis significantly predicted the burden imposed on the family caregivers (Mirsoleymani et al., 2017). Recent research on the burden on family caregivers of patients with cancer has yielded some intriguing results of the various factors contributing to caregiver burden. Some of these factors might be related to caregiver characteristics (e.g., age, gender, and relationship to the patient), while others may be related to patient characteristics (e.g., patient health status) and care-related activities (Maguire et al., 2018). It has been suggested that the factors such as worsening functional status of patients with cancer, younger age of the caregivers, being female, and longer caregiving durations are among the significant predictors of caregiving burden (Unsar et al., 2021).

A wide variety of factors might contribute to the cancer caregiver burden as multidimensional issues. Identifying such factors seems to be a key step for healthcare providers in managing the caregiving burden. However, there seems to be a paucity of data on the burden, anxiety, and depression among family caregivers of patients with cancer, as well as the contributing factors of such outcomes especially when it comes to low- and middle-income countries (Thrush and Hyder, 2014; Maguire et al., 2018). Therefore, we conducted a study to attain three objectives: prevalence of the caregiving burden, depression, and anxiety along with the relationships between aforementioned outcomes and the probable predicting factors for the caregiving burden among family caregivers of patients with cancer.

## Methods and materials

### Study design and participants

This was a cross-sectional study, conducted on a population of family caregivers of adult (≥18) patients with cancer at Vali-e-Asr Hospital in Zanjan, Iran from July 2019 to February 2020. We included participants aged ≥18 years who were the main family caregivers (unpaid and informal) of the patients with cancer. We defined main caregivers as those who had been providing care for the patient for at least 6 months and who had the most involvement in giving care for the patient and assisting them to adapt and manage the disease. In fact, they had been helping the patient in day-to-day activities such as feeding, relocation, psychological support, and emotional support, in addition to communicating with the healthcare team in relation to the patient's condition and medication.

Participants with a confirmed history of psychological or debilitating physical condition, as well as those unable to respond to the questionnaires, were excluded.

The study protocol was approved by the Ethics Committee of Zanjan University of Medical Sciences [IR.ZUMS.REC.1398.105]. Written informed consent was acquired from all participants after clarifying the purposes of the study. We adhered to the requirements of the Declaration of Helsinki.

### Measurements

Outcome variables, which included three variables of depression, anxiety, and caregiving burden, were measured by trained researchers using the Beck Depression Inventory (BDI-II), Beck Anxiety Inventory (BAI), and the Zarit Burden Interview (ZBI, 22-item), respectively. Furthermore, the data on variables such as gender, age, education, marital status, relationship to the patient under care, and duration of patient care as effect modifiers were collected using a questionnaire. In addition, patient-related data such as gender, stage of cancer, time since cancer diagnosis, care setting (inpatient or outpatient), type of treatment (radiation therapy or combination of radiation and chemotherapy), and Eastern Cooperative Oncology Group (ECOG) performance status were collected as confounding variables.

### Beck depression inventory (BDI-II)

BDI-II consists of 21 items, each scoring on a 4-point Likert scale from 0 to 3. Interpretation of the total score has been defined to be 10–13 for minimal depression, 14–19 for mild depression, 20–28 for moderate depression, and 29–63 for severe depression. Previous studies on the psychometric properties of this questionnaire in various countries have shown excellent validity. Wang et al. (2013) reported a high internal consistency reliability (Cronbach's α coefficient = 0.91) and a test–retest reliability of 0.93. According to a study conducted in Iran on non-clinical and clinical samples, internal consistency coefficients were reported to be 0.90 and 0.89, respectively. Additionally, the test–retest reliability coefficient has been shown to be 0.94 for the non-clinical sample (Ghassemzadeh et al., 2005). In this study, the Persian version of BDI-21 indicated excellent internal consistency with Cronbach's α coefficient of 0.92.

### Beck anxiety inventory (BAI)

This is a 21-item Likert-scale questionnaire designed by Beck and Steer (1990) to measure the anxiety of adults and adolescents. The total score of the questionnaire ranges between 0 and 63 in which minor, mild, moderate, and severe anxiety are represented by total scores of 0–7, 8–15, 16–25, and 26–63, respectively. de Beurs et al. (1997) obtained a Cronbach's α coefficient of 0.93 and a 5-week test–retest reliability coefficient of 0.83 for this questionnaire. A study on the Iranian population has shown Cronbach's α coefficient of 0.92 and a test–retest reliability of 0.83 (Rafiei and Seifi, 2013). In this study, the Persian version of BAI-21 demonstrated excellent internal consistency with Cronbach's α coefficient of 0.94.

### Zarit burden interview

ZBI initially consisted of 29 items; however, later in 2001, a shorter version, ZBI-22 with 12 questions and 4 questions was designed. ZBI-22 is rated on a 5-point Likert scale from 0 (never) to 4 (nearly always) for 21 first items and rated from 0 (not at all) to 4 (extremely) for the last item (total score, 0–88). Higher scores specify a greater burden on caregivers. ZBI-22 encompasses five domains of burden in the relationship (6 items), emotional wellbeing (7 items), social and family life (4 items), finances (1 item), and loss of control over one's life (4 items), which is designed to measure the perceived effect of caregiving on the caregiver's physical health, emotional health, social activities, and financial status (Boluarte-Carbajal et al., 2022). In fact, it evaluates the respondent's subjective burden by asking questions such as “Do you feel or do you wish ….” (Yu et al., 2020) Cronbach's α coefficient of ZBI-22 in caregivers of patients with cancer and dementia has been indicated to be in a range between 0.85 and 0.93 (Al-Rawashdeh et al., 2016). In this study, the Persian version of the 22-item of ZBI was applied. Navidian et al. (2010) have reported Cronbach's α coefficient of 0.91 and a test–retest reliability of 0.94 for ZBI-22 among Iranian subjects. In this study, the Persian version of ZBI showed good internal consistency with Cronbach's α coefficient of 0.88.

### Sample size

Regarding a caregiver burden of 87% (Mishra et al., 2021), sample size (*n*) was calculated to be at least 216 participants using the following formula: *n* = *Z*^2^ × *p* (1 – *p*)/*d*^2^, in which prevalence, *p* = 0.87; *Z* = 1.96; and margin of error, *d* = 0.05. However, we included more participants in the study. The participants were chosen by convenient sampling.

### Statistical analysis

Data were analyzed using SPSS software version 26. Descriptive statistics were reported using mean ± standard deviation (SD) and frequency (%), as applicable. To test the normality of data distribution, we used Shapiro–Wilk's test and the Box-Plot. For the purpose of comparing two groups in terms of outcome variables, we applied an independent samples *t*-test. An analysis of variance (ANOVA) was performed to compare ≥3 groups considering outcome variables with Tukey's honestly significant difference (HSD) *post-hoc* test if equal variances were assumed. In the instances where the assumption of equal variances was violated, Welch's ANOVA was used as an alternative to the Games–Howell *post-hoc* test. Pearson's correlation coefficient was conducted to examine the relationships between caregiving burden, depression, and anxiety. A stepwise linear regression analysis was done using dummy coded variables to investigate the role of demographic and basic characteristics of caregivers/patients as predictive variables on the outcomes of caregiving burden, anxiety, and depression among the family caregivers. The level of significance was considered 0.05 (two-sided) for all statistical analyses.

## Results

### Basic characteristics of the participants

A total of 300 family caregivers were included in the study. Of whom, 167 (55.7%) were males and 133 (44.3%) were females. The mean ± SD age of the caregivers was 40.77 ± 12.56 years. The majority of caregivers were offspring of the patients (148, 49.3%), married (239, 79.7%), and self-employed (81, 27.0%) (Table 1).

**Table 1:** Basic characteristics of the caregivers (*N* = 300).

**Caregivers**	**Mean ±SD/*N* (%)**
Age, years	40.77 ± 12.56
Gender, male	167 (55.7)
**Marital status**	
Married	239 (79.7)
Single	61 (20.3)
**Education**	
Illiterate	44 (14.7)
Primary school	44 (14.7)
Junior high school	37 (12.3)
Senior high school	11 (3.7)
HSD	77 (25.7)
Associate degree	27 (9.0)
BS	37 (12.3)
MSc and above	23 (7.7)
**Relationship to patient**	
Spouse	72 (24.0)
Offspring	148 (49.3)
Parents	38 (12.7)
Siblings	19 (6.3)
Others	23 (7.7)
**Employment status**	
Governmental employed	61 (20.3)
Self-employed	81 (27.0)
retired	53 (17.7)
unemployed	80 (26.7)
Quit for care	25 (8.3)
**Family income**	
≤ 40,000,000 IRR/month	87 (29.0)
40,000,000–80,000,000 IRR/month	99 (33.0)
≥80,000,000 IRR/month	114 (38.0)
**Presence of other caregivers**	
Yes	62 (20.8)
No	238 (79.3)
**Duration of caregiving, month**	
6–11	183 (61.0)
12–23	57 (19.0)
≥24	60 (20.0)

HSD, high school diploma; BS, Bachelor's degree; MSc, Master's degree.

With regard to the basic characteristics of the patients, as is shown in Table 2, they had an average age of 52.94 ± 14.33 and most of them were women (164, 54.7%). Stomach (61, 20.3%), lung (55, 18.3%), and colorectal (41, 13.7%) cancers were the most prevalent among patients. Most of them were under chemotherapy (151, 50.3%) and under chemotherapy + radiation therapy (74, 24.7%). Public health insurance (118, 39.3%) was the most common type of insurance applied by the patients.

**Table 2:** Basic characteristics of the patients.

**Patients**	**Mean ±SD/*N* (%)**
Age, years	52.94 ± 14.33
Gender, female	164 (54.7)
**Type of cancer**	
Breast	39 (13.0)
Prostate	34 (11.3)
Bladder	22 (7.3)
Stomach	61 (20.3)
Esophagus	30 (10.0)
Colorectal	41 (13.7)
Brain	18 (6.0)
Lung	55 (18.3)
**Stage of cancer**	
1	30 (10.0)
2	55 (18.3)
3	94 (31.3)
4	121 (40.3)
**ECOG**	
0	149 (49.7)
1	91 (30.3)
2	42 (14.0)
3	12 (4.0)
4	6 (2.0)
**Type of treatment**	
Chemo + radiation therapy	74 (24.7)
Radiation therapy	22 (7.3)
Surgery	31 (10.3)
Chemotherapy	151 (50.3)
Radio + hormone therapy	15 (5.0)
Chemo + hormone therapy	7 (5.0)
**Time since diagnosis, month**	
6–11	170 (56.7)
12–23	61 (20.3)
≥24	69 (23.0)
**Care setting**	
Inpatient	152 (50.7)
Outpatient	148 (49.3)
**Health insurance**	
Public health insurance	118 (39.3)
Social security insurance	98 (32.7)
Armed forces medical services insurance	19 (6.3)
supplemental insurance	65 (21.7)

ECOG, functional performance status.

### Depression, anxiety, and burden among family caregivers

**Figure 1:**
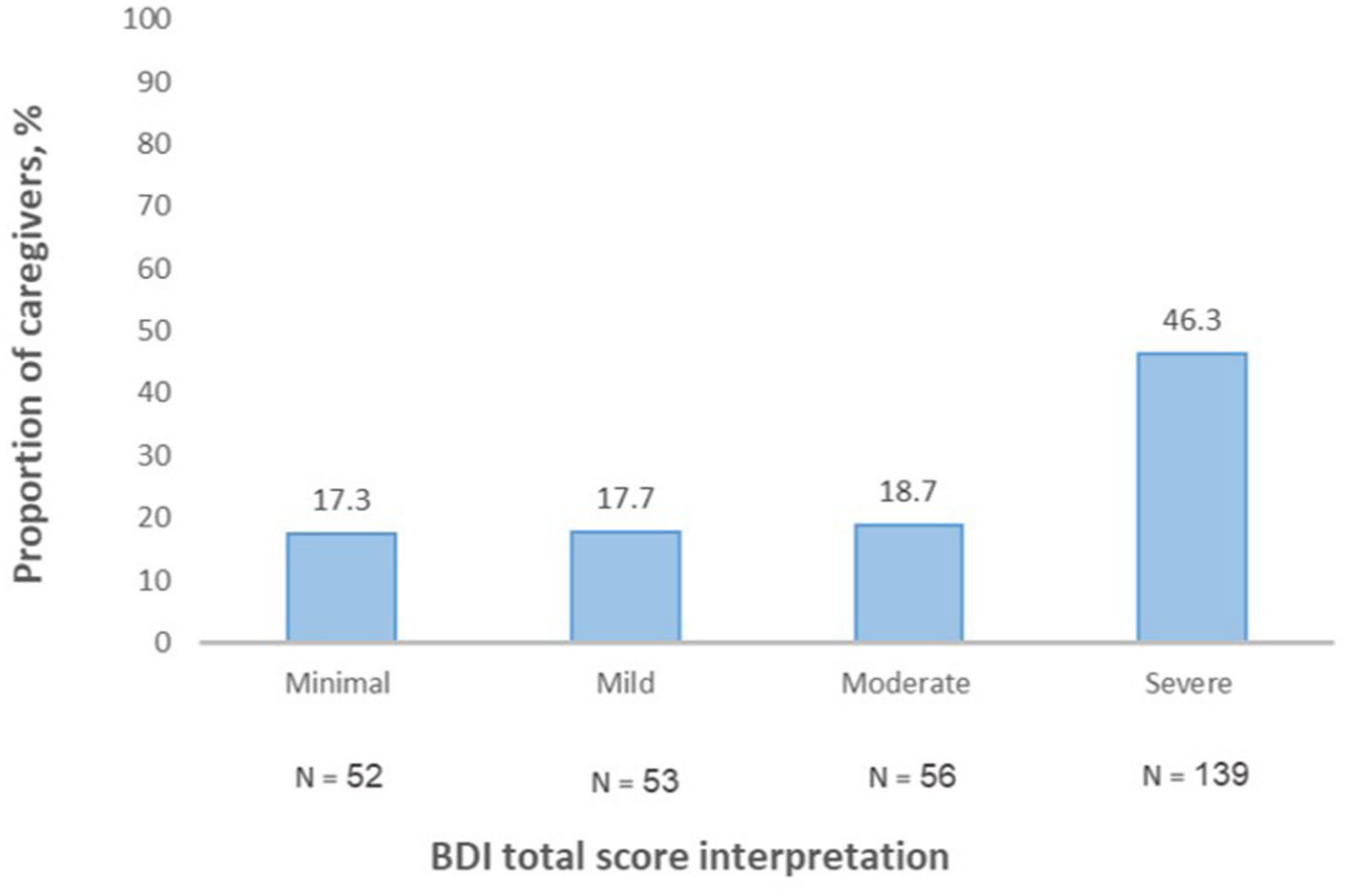
Beck Depression Inventory (BDI-II) total score interpretation.

Considering the level of depression among the caregivers, BDI total score mean was 28.01 ± 13.28. The BDI total score interpretation revealed that 52 (17.3%) caregivers had minimal or no depression (total score, 0–13), 53 (17.7%) had mild (total score, 4–19), 56 (18.7%) had moderate (total score, 20–28), and 139 (46.3%) had severe depression (total score, 29–63) (Figure 1).

**Figure 2:**
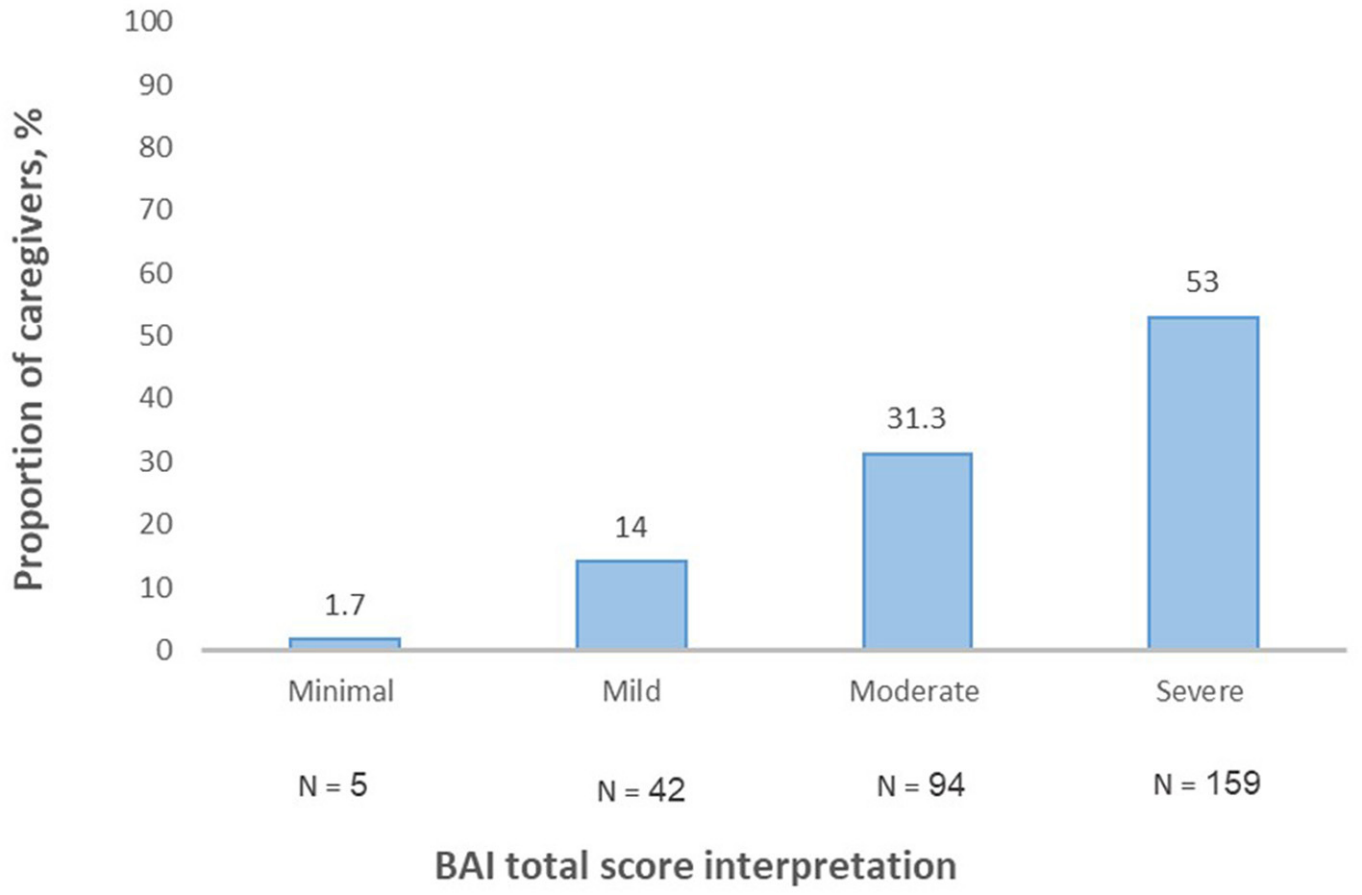
Beck Anxiety Inventory (BAI) total score interpretation.

The BAI's total score mean was found to be 31.49 ± 13.87. Almost half of the caregivers represented severe anxiety (159, 53%) (Figure 2).

**Figure 3:**
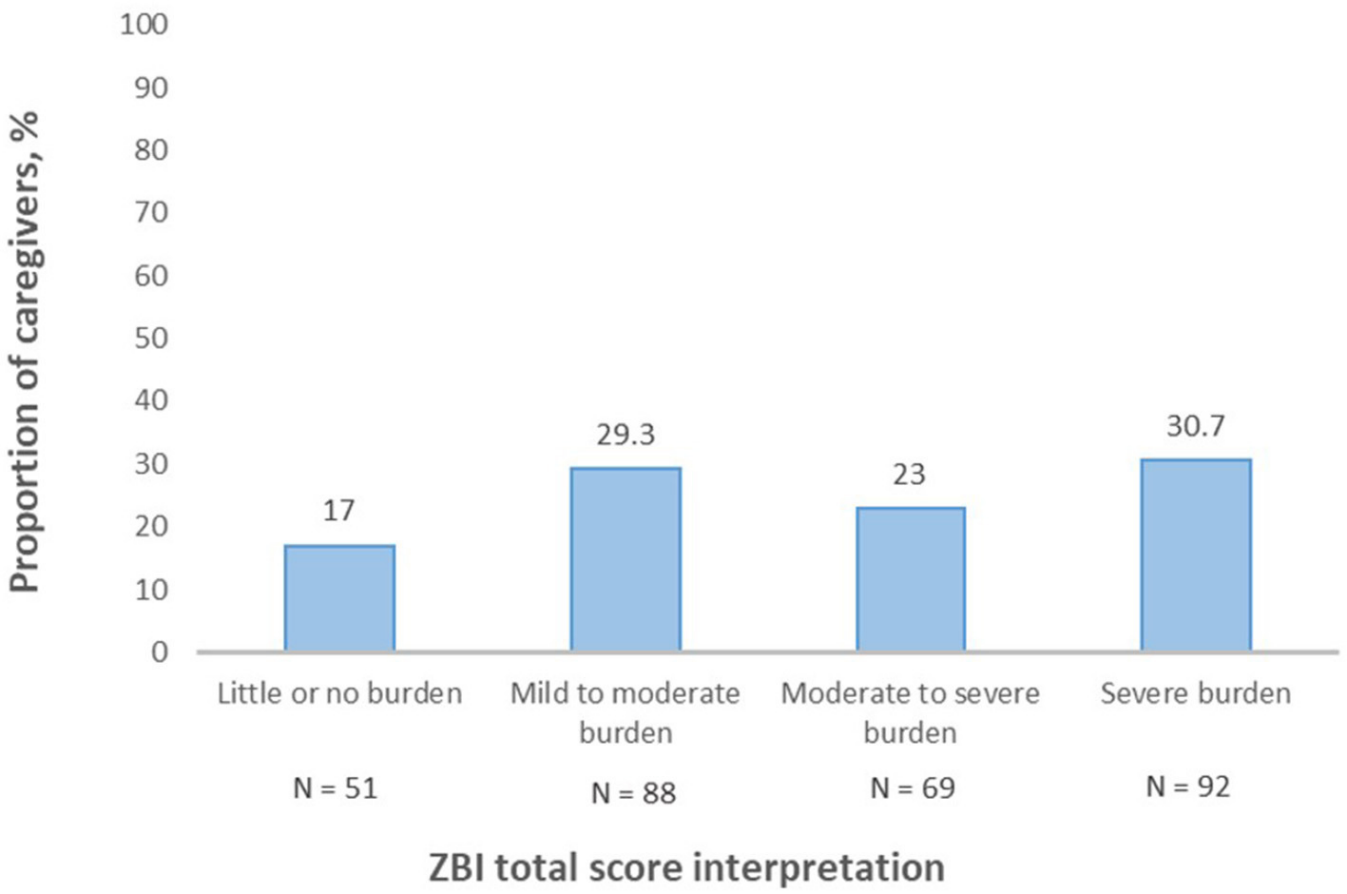
Zarit Burden Interview (ZBI) total score interpretation.

The ZBI's total score mean was 54.75 ± 17.86. Severe burden (92, 30.7%) was the most frequent category among caregivers (Figure 3).

Then, we conducted an ANOVA test to examine the effects of participants' basic characteristics on the level of depression, anxiety, and burden among family caregivers.

### BDI

Relationship to the patient showed a significant effect on the level of depression for the five conditions [*F*_(4, 295)_ = 10.576, *p* < 0.001], so that, the mean score for being a parent (*M* = 33.76, SD = 14.92) was significantly different from being an offspring (*M* = 24.68, SD = 11.70, *p* = 0.009) and others (*M* = 20.39, SD = 10.88, *p* = 0.002). Moreover, being a spouse (*M* = 33.65, SD = 13.07) represented a significantly different mean level of total BDI from those who were either offspring or others to the patient (both, *p* < 0.001).

The difference between the five age categories of the patients was found to be statistically significant regarding the level of caregiver's depression [*F*_(4, 295)_ = 4.070, *p* = 0.003]. A *post-hoc* test showed that caregiving for a young patient (≤ 30) caused a significantly more level of depression than the other age ranges (all, *p* < 0.05).

There was a significant difference between various types of patient treatments [*F*_(5, 294)_ = 2.161, *p* = 0.022]. The Games–Howell *post-hoc* test comparisons uncovered that the mean BDI score for the surgery (*M* = 21.06, SD = 9.91) was significantly lower than the other treatments (all, *p* < 0.05).

The mean BDI score for patients with social security insurance was significantly higher than that among those who had Armed Forces Medical Services Insurance (21.37 vs. 31.38, *p* = 0.013). None of the other comparisons were significant.

None of the other variables showed a significant difference (all, *p* ≥ 0.05) (Table 3).

**Table 3:** Comparison of BDI total score in terms of basic characteristics of the family caregivers and patients.

**Caregiver**	**Mean ±SD**	**CI 95%/MD (CI 95%)**	**F/t**	***P*-value**	**Patients**	**Mean ±SD**	**CI 95%/MD (CI 95%)**	**F/t**	***P*-value**
**Gender**					**Gender**				
Female	28.65 ± 12.86	−1.15 (−4.19, 1.89)	−0.744	0.457^†^	Female	28.48 ± 12.47	−1.03 (−4.06, 2.00)	−0.671	0.503^†^
Male	27.50 ± 13.62				Male	27.44 ± 14.21			
**Age, year**					**Age, year**				
≤ 30	27.71 ± 12.70	24.56, 30.85	0.506	0.731^*^	≤ 30	36.11 ± 14.91	30.99, 41.24	4.070	**0.003** ^*^
31–40	27.22 ± 13.93	24.30, 30.14			31–40	25.89 ± 13.36	21.37, 30.41		
41–50	29.99 ± 14.11	26.54, 33.43			41–50	26.39 ± 12.97	23.01, 29.77		
51–60	26.97 ± 13.30	22.17, 31.77			51–60	28.20 ± 12.58	24.97, 31.42		
≥61	27.80 ± 11.68	24.33, 31.27			≥61	26.87 ± 12.51	24.50, 29.25		
**Marital status**					**Care setting**				
Married	28.11 ± 13.50	−0.52 (−4.27, 3.23)	−0.274	0.784^†^	Inpatient	28.34 ± 13.08	−0.667 (−2.35, 3.68)	−0.434	0.665^†^
Single	27.59 ± 12.46				Outpatient	27.67 ± 13.51			
**Education**					**Type of cancer**				
Illiterate	29.73 ± 12.87	25.81, 33.64	1.183	0.312^*^	Breast	25.54 ± 13.20	21.26, 29.82	1.179	0.315^*^
Primary school	28.52 ± 12.68	24.67, 32.38			Prostate	23.97 ± 13.93	19.11, 28.83		
Junior high school	30.14 ± 12.77	25.88, 34.39			Bladder	28.36 ± 14.03	22.14, 34.59		
Senior high school	26.00 ± 13.57	16.88, 35.12			Stomach	27.59 ± 12.50	24.39, 30.79		
HSD	26.21 ± 13.54	23.13, 29.28			Esophagus	27.97 ± 12.32	23.37, 32.57		
Associate degree	29.56 ± 11.52	25.00, 34.11			Colorectal	29.41 ± 13.40	25.18, 33.65		
BS	29.78 ± 14.71	24.88, 34.69			Brain	32.06 ± 16.07	24.06, 40.05		
MSc and above	22.61 ± 14.06	16.53, 28.69			Lung	30.22 ± 12.74	26.77, 33.66		
**Relationship to patient**					**Stage of cancer**				
Spouse	33.65 ± 13.07	30.58, 36.72	10.576	**<0.001** ^‡^	1	23.50 ± 10.74	19.49, 27.51	1.52	0.207^*^
Offspring	24.68 ± 11.70	22.78, 26.58			2	27.15 ± 13.04	23.62, 30.67		
Parents	33.76 ± 14.92	28.86, 38.67			3	28.88 ± 13.19	26.18, 31.59		
Siblings	30.21 ± 12.96	23.96, 36.46			4	28.83 ± 13.90	26.33, 31.34		
Others	20.39 ± 10.88	15.69, 25.10			**ECOG performance status**				
**Employment status**					0	27.52 ± 13.33	25.36, 29.68	0.483	0.748^*^
Governmental employed	26.89 ± 14.29	23.22, 30.55	0.627	0.644^*^	1	27.49 ± 12.36	24.92, 30.07		
Self-employed	29.30 ± 13.65	26.28, 32.32			2	29.86 ± 14.53	25.33, 34.39		
Retired	27.13 ± 12.93	23.57, 30.70			3	31.42 ± 14.73	22.05, 40.78		
Unemployed	27.35 ± 12.02	24.67, 30.03			4	28.17 ± 16.00	11.37, 44.96		
Quit for care	30.52 ± 14.46	24.55, 36.49			**Time since diagnosis, month**				
**Family income**					6–11	27.69 ± 13.11	25.70, 29.67	0.118	0.889^*^
≤ 40,000,000 IRR/month	26.26 ± 12.06	23.69, 28.84	1.311	0.271^*^	12–23	28.56 ± 13.16	25.19, 31.93		
40,000,000–80,000,000 IRR/month	29.41 ± 13.38	26.74, 32.08			≥24	28.30 ± 13.95	24.95, 31.66		
≥80,000,000 IRR/month	28.11 ± 14.02	25.51, 30.72			**Type of treatment**				
**Presence of other caregivers**					Chemo + Radiation therapy	29.92 ± 14.31	26.60, 33.24	2.161	**0.022** ^‡^
Yes	28.48 ± 12.95	−0.60 (−4.33, 3.13)	−0.317	0.751^†^	Radiation therapy	29.09 ± 15.25	22.33, 35.85		
No	27.88 ± 13.39				Surgery	21.06 ± 9.91	17.43, 24.70		
**Duration of caregiving, month**					Chemotherapy	28.07 ± 12.29	26.09, 30.04		
6–11	27.28 ± 13.17	25.36, 29.20	0.954	0.386^*^	Radio + Hormone therapy	29.60 ± 15.16	21.20, 38.00		
12–23	30.04 ± 12.77	26.64, 33.43			Chemo + Hormone therapy	30.43 ± 19.04	12.82, 48.04		
≥24	28.30 ± 14.06	1.816, 24.67			**Health insurance**				
					Public health insurance	26.86 ± 13.87	24.34, 29.39	4.251	**0.006** ^*^
					Social security insurance	31.38 ± 12.26	28.92, 33.84		
					Armed forces medical services insurance	21.37 ± 10.72	16.20, 26.54		
					Supplemental insurance	26.94 ± 13.35	23.63, 30.25		

* P < 0.05, obtained from ANOVA F-test.

† P < 0.05, obtained from independent samples t-test.

‡ P < 0.05, obtained from Welch's ANOVA.

Significant P-values are shown in bold.

HSD, high school diploma; BS, bachelor's degree; MS, master's degree.

### BAI

Female caregivers (*M* = 33.44, SD = 13.66) in comparison with the male caregivers (*M* = 29.93, SD = 13.89) demonstrated significantly higher levels of anxiety, *t*(298) = −2.185, *p* = 0.030.

Different conditions of the “relationship to the patient” variable differed significantly in terms of BAI total score [*F*_(4, 295)_ = 3.062, *p* = 0.017] although there were no significant differences in multiple comparisons using Tukey's HSD (all, *p* ≥ 0.05).

The level of anxiety was significantly different between the three conditions of “duration of caregiving” [*F*_(2, 297)_ = 7.206, *p* = 0.001]. Being a caregiver for ≥24 months resulted in a significantly greater level of anxiety compared to the duration of 6–11 months (mean, 37.22 vs. 29.54, *p* = 0.001).

Surprisingly, caring for female patients was also associated with higher levels of anxiety compared to male patients, *t*(298) = −2.162, *p* = 0.031.

There was a significant difference between age categories of patients regarding the caregivers' level of anxiety (*p* = 0.036) so the Games–Howell *post-hoc* test revealed that caring for patients ≤ 30 years was significantly linked with higher levels of anxiety in comparison with patients ≥61 years (mean, 37.89 vs. 29.33, *p* = 0.043).

The mean score of total BAI showed a significant difference between different types of cancer [*F*_(7, 292)_ = 3.133, *p* = 0.003]. Caregivers of patients with prostate cancer (*M* = 24.41, SD = 14.18) demonstrated significantly lower levels of anxiety compared to those who care for patients with lung (*M* = 35.53, SD = 13.73, *p* = 0.005) and brain (*M* = 37.89, SD = 14.99, *p* = 0.017) cancers.

Caring for patients with stage 1 cancer (*M* = 24.27, SD = 13.12) was significantly associated with lower levels of anxiety [*F*_(3, 296)_ = 3.122, *p* = 0.026], as opposed to stage 2 (*M* = 32.80, SD = 14.81), stage 3 (*M* = 32.41, SD = 13.49), stage 4 (*M* = 31.96, SD = 13.55).

Time since diagnosis showed a significant effect on the level of anxiety for the three conditions [*F*_(2, 297)_ = 10.383, *p* < 0.001]. Caring for patients with ≥24 months from their diagnosis was significantly related to more levels of anxiety in contrast to those with either 6–11 months (*M* = 29.69, SD = 13.59) or 12–23 months (*M* = 29.16, SD = 13.55).

The mean total score of BAI was significantly different between different types of treatment (*p* = 0.003) so the Games–Howell *post-hoc* test demonstrated that being treated by surgery was associated with a lower level of anxiety among caregivers compared to chemotherapy + hormone (*p* = 0.043) and chemotherapy + radiation therapy (*p* = 0.001).

Caregivers also experienced significantly different levels of anxiety based on the types of health insurance, even though multiple comparisons were not significant (all, *p* ≥ 0.05).

None of the other variables showed a significant difference (all, *p* ≥ 0.05) (Table 4).

**Table 4:** Comparison of BAI total score in terms of basic characteristics of the family caregivers and patients.

**Caregiver**	**Mean ±SD**	**CI 95%/MD (CI 95%)**	**F/t**	***P*-value**	**Patients**	**Mean ±SD**	**CI 95%/MD (CI 95%)**	**F/t**	***P*-value**
**Gender**					**Gender**				
Female	33.44 ± 13.66	−3.50 (−6.65, −0.34)	−2.185	**0.030** ^†^	Female	33.05 ± 13.58	−3.45 (−6.60, −0.31)	−2.162	**0.031** ^†^
Male	29.93 ± 13.89				Male	29.60 ± 14.04			
**Age, year**					**Age, year**				
≤ 30	31.68 ± 13.21	28.40, 34.95	1.240	0.294^*^	≤ 30	37.89 ± 15.83	32.44, 43.33	3.150	**0.036** ^‡^
31–40	28.97 ± 13.91	26.05, 31.88			31–40	32.81 ± 14.86	27.77, 37.84		
41–50	33.48 ± 13.95	30.07, 36.88			41–50	29.46 ± 13.30	25.99, 32.93		
51–60	33.25 ± 13.26	28.47, 38.03			51–60	32.85 ± 12.71	29.60, 36.11		
≥61	32.02 ± 14.86	27.61, 36.44			≥61	29.33 ± 13.26	26.81, 31.85		
**Marital status**					**Care setting**				
Married	31.55 ± 14.01	−0.32 (−4.24, 3.60)	−0.161	0.872^†^	Inpatient	32.22 ± 13.54	−1.49 (−1.66, 4.64)	−0.932	0.352^†^
Single	31.23 ± 13.44				Outpatient	30.73 ± 14.21			
**Education**					**Type of cancer**				
Illiterate	33.23 ± 14.28	28.88, 37.57	0.372	0.918^*^	Breast	31.00 ± 14.61	26.26, 35.74	3.133	**0.003** ^*^
Primary school	32.30 ± 11.78	28.71, 35.88			Prostate	24.41 ± 14.18	19.46, 29.36		
Junior high school	31.27 ± 14.28	26.51, 36.03			Bladder	35.23 ± 13.58	29.20, 41.25		
Senior high school	30.36 ± 14.72	20.47, 40.26			Stomach	29.05 ± 12.85	25.76, 32.34		
HSD	30.12 ± 14.44	26.84, 33.40			Esophagus	31.03 ± 11.52	26.73, 35.34		
Associate degree	33.70 ± 13.95	28.19, 39.22			Colorectal	31.54 ± 13.38	27.31, 35.76		
BS	31.16 ± 14.03	26.48, 35.84			Brain	37.89 ± 14.99	30.43, 45.34		
MSc and above	30.00 ± 14.79	23.60, 36.40			Lung	35.53 ± 13.73	31.81, 39.24		
**Relationship to patient**					**Stage of cancer**				
Spouse	34.50 ± 13.64	31.29, 37.71	3.062	**0.017** ^*^	1	24.27 ± 13.12	19.37, 29.17	3.122	**0.026** ^*^
Parents	34.79 ± 14.81	29.92, 39.66			2	32.80 ± 14.81	28.80, 36.80		
Offspring	29.65 ± 12.95	27.54, 31.75			3	32.41 ± 13.49	29.65, 35.18		
Siblings	34.00 ± 15.98	26.30, 41.70			4	31.96 ± 13.55	29.52, 34.40		
Others	26.35 ± 14.63	20.02, 32.68			**ECOG performance status**				
**Employment status**					0	30.97 ± 13.59	28.77, 33.17	1.769	0.135^*^
Governmental employed	31.10 ± 14.35	27.42, 34.78	0.218	0.928^*^	1	32.69 ± 13.94	29.79, 35.60		
Self-employed	31.21 ± 13.32	28.26, 34.16			2	33.64 ± 14.45	29.14, 38.15		
Retired	32.64 ± 15.44	28.38, 36.90			3	22.67 ± 12.61	14.65, 30.68		
Unemployed	30.85 ± 13.54	27.84, 33.86			4	28.67 ± 14.77	13.16, 44.17		
Quit for care	32.92 ± 12.82	27.62, 38.22			**Time since diagnosis, month**				
**Family income**					6–11	29.69 ± 13.59	27.64, 31.75	10.383	**≤0.001** ^*^
≤ 40,000,000 IRR/month	30.87 ± 13.54	27.99, 33.76	0.394	0.675^*^	12–23	29.16 ± 13.55	25.69, 32.63		
40,000,000–80,000,000 IRR/month	32.49 ± 14.04	29.69, 35.30			≥24	37.96 ± 13.05	34.82, 41.09		
≥80,000,000 IRR/month	31.08 ± 14.06	28.47, 33.69			**Type of treatment**				
**Presence of other caregivers**					Chemo + Radiation therapy	35.78 ± 14.71	32.37, 39.19	4.282	**0.003** ^‡^
Yes	33.16 ± 13.93	−2.11 (−6.00, 1.78)	−1.067	0.287^†^	Radiation therapy	29.14 ± 17.10	21.55, 36.72		
No	31.05 ± 13.86				Surgery	24.06 ± 11.39	19.88, 28.25		
**Duration of caregiving, month**					Chemotherapy	31.10 ± 12.65	29.06, 33.13		
6–11	29.54 ± 13.69	27.54, 31.54	7.206	**0.001** ^*^	Radio + Hormone therapy	28.73 ± 13.96	21.00, 36.46		
12–23	31.70 ± 13.58	28.10, 35.31			Chemo + Hormone therapy	40.57 ± 13.39	28.19, 52.95		
≥24	37.22 ± 13.32	33.78, 40.66			**Health insurance**				
					Public health insurance	30.40 ± 13.83	27.88, 32.92	3.263	**0.022** ^*^
					Social security insurance	34.79 ± 13.63	32.05, 37.52		
					Armed forces medical services insurance	26.42 ± 14.38	19.49, 33.36		
					Supplemental insurance	29.97 ± 13.46	26.63, 33.31		

* P < 0.05, obtained from ANOVA F-test.

† P < 0.05, obtained from independent samples t-test.

‡ P < 0.05, obtained from Welch's ANOVA.

Significant P-values are shown in bold.

HSD, high school diploma; BS, bachelor's degree; MS, master's degree; SD, standard deviation, MD, mean difference.

### ZBI

Female caregivers experienced a significantly higher burden than males [mean ± SD, 57.14 ± 17.13 vs. 52.84 ± 18.25, *t*(298) = −2.078, *p* = 0.039].

The total score of ZBI was significantly different between age groups of caregivers [*F*_(4, 295)_ = 7.280, *p* < 0.001]. Being a caregiver ≥61 years was significantly associated with higher burdens compared to those ≤ 30 (*p* = 0.001), 31–40 (*p* = 0.001), and 41–50 (*p* < 0.001) years. The age group of 51–60 years also showed a significant difference in comparison with the age group of 41–50 years (*p* = 0.027).

The total score of ZBI significantly differed among caregiver's levels of education [*F*_(7, 292)_ = 2.955, *p* = 0.005] so that being illiterate was significantly linked with higher levels of the burden against those with junior high school (*p* = 0.007), high school diploma (HSD) (*p* = 0.005), and master of science (MSc) (*p* = 0.018) degrees.

Regarding employment status, the total score of ZBI was significantly different among different conditions (*p* < 0.001). A Games–Howell *post-hoc* test revealed that being a retired caregiver was significantly related to a greater burden as opposed to being government-employed (*p* < 0.001), self-employed (*p* < 0.001), and unemployed (*p* < 0.001).

There was a significant association between the duration of caregiving and ZBI [*F*_(2, 297)_ = 24.564, *p* < 0.001]. Being in the role of a caregiver for ≥24 months was significantly related to higher levels of burden compared to 12–23 (*p* < 0.001) and 6–11 months (*p* < 0.001).

Similarly, time since the diagnosis also showed a significant effect on the level of burden [*F*_(2, 297)_ = 20.217, *p* < 0.001]. Caregiving for a patient diagnosed ≥24 months was associated with higher levels of burden compared to 12–23 (*p* = 0.001) and 6–11 months (*p* < 0.001).

Caregivers of inpatients experienced significantly more levels of burden in comparison with outpatients [*t*(298) = 5.924, *p* < 0.001].

We found a significant effect of health insurance type on the level of burden (*p* < 0.001). The Games–Howell *post-hoc* test revealed that caregivers of patients with supplemental insurance experienced lower levels of burden compared to those with public health (*p* = 0.001) and social security (*p* < 0.001) insurance.

No significant associations were found between other basic characteristics of the participants and ZBI (all, *p* ≥ 0.05) (Table 5).

**Table 5:** Comparison of ZBI total score in terms of basic characteristics of the family caregivers and patients.

**Caregiver**	**Mean ±SD**	**CI 95%/MD (CI 95%)**	**F/t**	***P*-value**	**Patients**	**Mean ±SD**	**CI 95%/MD (CI 95%)**	**F/t**	***P*-value**
**Gender**					**Gender**				
Female	57.14 ± 17.13	−4.29 (−8.35, −0.22)	−2.078	**0.039** ^†^	Female	56.21 ± 17.63	−3.22 (−7.29, 0.84)	−1.558	0.120^†^
Male	52.84 ± 18.25				Male	52.99 ± 18.05			
**Age, year**					**Age, year**				
≤ 30	52.22 ± 18.49	47.63, 56.80	7.280	**<0.0001** ^*^	≤ 30	52.71 ± 18.88	46.23, 59.20	0.875	0.480
31–40	52.84 ± 17.28	49.23, 56.46			31–40	55.67 ± 17.85	49.63, 61.71		
41–50	49.75 ± 17.36	45.51, 53.98			41–50	51.61 ± 19.32	46.57, 56.65		
51–60	60.66 ± 16.98	54.53, 66.78			51–60	55.20 ± 17.04	50.83, 59.56		
≥61	65.22 ± 14.59	60.88, 69.55			≥61	56.54 ± 17.20	53.27, 59.81		
**Marital status**					**Care setting**				
Married	55.76 ± 17.58	−4.99 (−10.01, 0.03)	−1.956	0.051^†^	Inpatient	60.46 ± 15.30	−11.58 (−7.73, −15.43)	−5.924	**<0.0001** ^†^
Single	50.77 ± 18.57				Outpatient	48.88 ± 18.44			
**Education**					**Type of cancer**				
Illiterate	64.89 ± 16.25	59.95, 69.83	2.955	**0.005** ^*^	Breast	58.13 ± 17.44	52.47, 63.78	1.460	0.181^*^
Primary school	55.36 ± 16.53	50.34, 60.39			Prostate	50.62 ± 16.87	44.73, 56.51		
Junior high school	50.59 ± 15.89	45.30, 55.89			Bladder	60.68 ± 15.65	53.74, 67.62		
Senior high school	55.27 ± 16.12	44.44, 66.11			Stomach	56.95 ± 17.31	52.52, 61.39		
HSD	52.48 ± 18.33	48.32, 56.64			Esophagus	50.57 ± 17.88	43.89, 57.25		
Associate degree	52.59 ± 16.74	45.97, 59.22			Colorectal	53.12 ± 19.80	46.87, 59.37		
BS	55.41 ± 18.62	49.20, 61.61			Brain	49.56 ± 19.41	39.90, 59.21		
MSc and above	49.65 ± 20.25	40.90, 58.41			Lung	55.27 ± 17.61	50.51, 60.03		
**Relationship to patient**					**Stage of cancer**				
Spouse	58.81 ± 16.14	55.01, 62.60	1.398	0.234^*^	1	52.57 ± 16.17	46.53, 58.61	0.210	0.889^*^
Parents	53.53 ± 16.66	48.05, 59.00			2	54.20 ± 18.94	49.08, 59.32		
Offspring	53.11 ± 18.68	50.07, 56.14			3	55.10 ± 18.40	51.33, 58.87		
Siblings	52.68 ± 19.11	43.47, 61.90			4	55.26 ± 17.51	52.11, 58.42		
Others	56.30 ± 17.86	48.58, 64.03			**ECOG Performance Status**				
**Employment status**					0	54.30 ± 18.00	51.39, 57.22	0.438	0.781^*^
Governmental employed	51.31 ± 16.58	47.06, 55.56	8.417	**<0.0001** ^‡^	1	56.04 ± 17.89	52.32, 59.77		
Self-employed	50.36 ± 17.23	46.55, 54.17			2	55.29 ± 18.85	49.41, 61.16		
Retired	66.42 ± 14.61	62.39, 70.44			3	51.92 ± 12.24	44.14, 59.70		
Unemployed	53.36 ± 17.45	49.48, 57.25			4	48.00 ± 19.47	27.56, 68.44		
Quit for care	57.04 ± 20.67	48.50, 65.58			**Time since diagnosis, month**				
**Family income**					6–11	50.34 ± 15.98	47.92, 52.76	20.217	**<0.0001** ^*^
≤ 40,000,000 IRR/month	57.84 ± 20.83	53.40, 62.28	3.060	0.056^‡^	12–23	54.74 ± 17.95	50.14, 59.34		
40,000,000–80,000,000 IRR/month	55.51 ± 16.25	52.26, 58.75			≥24	65.61 ± 17.77	61.34, 69.88		
≥80,000,000 IRR/month	51.73 ± 16.38	48.69, 54.77			**Type of treatment**				
**Presence of other caregivers**					Chemo + Radiation therapy	53.72 ± 18.55	49.42, 58.01	0.907	0.476^*^
Yes	55.31 ± 17.48	−0.70 (−5.72, 4.31)	−0.277	0.782^†^	Radiation therapy	53.55 ± 18.23	45.46, 61.63		
No	54.60 ± 18.00				Surgery	53.77 ± 16.69	47.65, 59.90		
**Duration of caregiving, month**					Chemotherapy	56.32 ± 17.93	53.44, 59.21		
6–11	50.31 ± 15.94	47.98, 52.63	24.564	**<0.0001** ^*^	Radio + Hormone therapy	46.93 ± 16.59	37.74, 56.12		
12–23	55.47 ± 18.67	50.52, 60.43			Chemo + Hormone therapy	56.43 ± 15.50	42.09, 70.76		
≥24	67.60 ± 16.52	63.33, 71.87			**Health insurance**				
					Public health insurance	55.04 ± 19.44	51.50, 58.59	5.447	**<0.0001** ^‡^
					Social security insurance	58.47 ± 17.20	55.02, 61.92		
					Armed forces medical services insurance	58.37 ± 15.51	50.89, 65.85		
					Supplemental insurance	47.54 ± 14.35	43.98, 51.09		

* P <0.05, obtained from ANOVA F-test.

† P <0.05, obtained from independent samples t-test.

‡ P <0.05, obtained from Welch's ANOVA.

Significant P-values are shown in bold.

HSD, high school diploma; BS, bachelor's degree; MS, master's degree; SD, standard deviation; MD, mean difference.

### Bivariate correlations between all outcomes

Results of the Pearson correlation demonstrated that there was a significant positive correlation between ZBI with both BDI [*r*_(298)_ = 0.19, *p* < 0.01] and BAI [*r*_(298)_ = 0.20, *p* < 0.01] (Table 6).

**Table 6:** Bivariate correlations between all outcomes (*N* = 300).

		**(1)**	**(2)**	**(3)**
(1)	Depression	1		
(2)	Anxiety	0.612^**^	1	
(3)	Burden	0.196^**^	0.201^**^	1

** Correlation is significant at the 0.01 level (2-tailed).

### Predicting factors for caregiver burden

A stepwise linear regression was performed to predict caregiver burden based on the variables of basic characteristics, depression, and anxiety of the participants. Regression analysis resulted in nine significant models (Table 7).

**Table 7:** Predicting factors for caregiver burden according to stepwise linear regression models (*N* = 300).

**Independent variables**	**Model 1**	**Model 2**	**Model 3**	**Model 4**	**Model 5**	**Model 6**	**Model 7**	**Model 8**	**Model 9**
*F*-value	44.44	45.06	43.53	38.53	34.13	30.16	27.85	25.65	23.51
Corrected *R*-squared	0.12	0.22	0.29	0.33	0.35	0.36	0.38	0.39	0.40
Constants	51.53^***^	68.66^***^	66.32^***^	58.77^***^	59.79^***^	60.05^***^	60.43^***^	60.22^***^	53.43^***^
(1) Duration of caregiving (≥24 months)	16.06^***^	15.92^***^	15.02^***^	14.91^***^	14.96^***^	14.99^***^	14.84^***^	14.63^***^	14.361^***^
(2) Care setting (outpatient)		−11.45^***^	−11.26^***^	−11.09^***^	−10.56^***^	−10.82^***^	−12.13^***^	−13.07^***^	−12.90^***^
(3) Employment status (retired)			12.70^***^	12.99^***^	12.45^***^	9.56^***^	8.80^***^	9.13^***^	9.48^***^
(4) Depression				0.25^***^	0.25^***^	0.23^***^	0.26^***^	0.27^***^	0.28^***^
(5) Health insurance (supplemental)					−6.77^**^	−6.89^**^	−7.79^***^	−7.31^***^	−7.79^***^
(6) Education (illiterate)						6.84^**^	8.27^**^	8.32^**^	7.77^**^
(7) Type of treatment (surgery)							8.61^**^	8.51^**^	8.55^**^
(8) ECOG (1)								4.60	4.88^**^
(9) Patients' age									0.11^*^

^*^p <0.05, ^**^p <0.01, ^***^p <0.001.

Duration of caregiving (≥24 months), care setting (outpatient), employment status (retired), depression, health insurance (supplemental), education (illiterate), type of treatment (surgery), ECOG (1), and patient's age were significant predictors of caregiver burden.

The results of the first model were found to be statistically significant (*p* < 0.001), suggesting duration of caregiving (≥24 months) is a significant predictor of caregiver burden. According to the *R*^2^-value (*R*^2^ = 0.12) associated with this model, the duration of caregiving (≥24 months) accounts for 12% of the variation in caregiver burden, which means that 88% of the variation in the burden cannot be explained by the duration of caregiving (≥24 months) alone. The regression coefficient [*B* = 16.06, 95% confidence interval (CI): 11.32–20.81, *p* < 0.001] associated with duration of caregiving showed that caregiving for patients ≥24 months resulted in 16.06 units more burden than either 6–11 or 12–23 months.

The second model was also statistically significant (*p* < 0.001) for which, care setting (outpatient) was added to the analysis. The *R*^2^-value (*R*^2^ = 0.22) associated with this model indicates that the addition of care setting to the first model accounts for 22% of the variation in caregiver burden, which means that 78% of the variation in the burden cannot be explained by the duration of caregiving and care setting alone. Controlling for care setting, the regression coefficient (*B* = 15.92, 95% CI: 11.46–20.38, *p* < 0.001) associated with duration of caregiving demonstrated that caregiving for patients ≥24 months resulted in 15.92 units more burden than either 6–11 or 12–23 months. Controlling for the duration of caregiving, the regression coefficient (*B* = −11.45, 95% CI: −15.02 to −7.88, *p* < 0.001) associated with care setting revealed that caregivers of outpatients experienced 11.45 units lower levels of burden than those of inpatients. Table 7 shows all nine regression models in detail.

We tested the data to explore whether the assumption of collinearity was met or not met. The results indicated that multicollinearity was not a concern (caregiving ≥24 months, tolerance = 0.98, variance inflation factor (VIF) = 1.01, outpatient care setting, tolerance = 0.87, VIF = 1.14; being retired, tolerance = 0.76, VIF = 1.30; depression, tolerance = 0.94, VIF = 1.05; supplemental health insurance, tolerance = 0.93, VIF = 1.06; being illiterate, tolerance = 0.75, VIF = 1.32; surgery, tolerance = 0.85, VIF = 1.16; ECOG1, tolerance = 0.93, VIF = 1.07; and patients' age, tolerance = 0.94, VIF = 1.05).

The data also met the assumption of independent errors (Durbin–Watson value = 1.92).

## Discussion

In this study, three key results were established. First, our findings demonstrated a noticeable prevalence of depression, anxiety, and high burden among family caregivers. Second, the caregiving burden was positively correlated with both depression and anxiety. Finally, nine significant variables were suggested for predicting the caregiving burden.

With regard to psychological consequences, a considerable number of cancer caregivers have been discovered to be positive for anxiety and depression screening (Sklenarova et al., 2015). As studied by Götze et al. (2018), a significant proportion of cancer caregivers showed severe symptoms of anxiety (32%) and depression (29%). According to a systematic review, the prevalence of depression and anxiety among the population of cancer caregivers was found to be 42.30 and 46.56%, respectively (Geng et al., 2018). As indicated by this study, almost half of the caregivers showed severe anxiety (53%) and depression (46.3%).

This notable prevalence of psychological consequences can be caused by the challenges family caregivers have to face and the painful realities they should accept. One qualitative study has inferred that the major worry of the family caregivers was the gradual weariness of their patients and the fact that they are on the edge of their impending death (Taleghani et al., 2021). In fact, the family caregivers are likely to suffer immense psychological distress from the time of cancer diagnosis to the last moment of their patient's life. However, it has been stated that the anxiety and depression of cancer caregivers who were grieved at losing their loved one had lessened significantly although extreme depressive symptoms remained among 25.0% of the bereaved (Sklenarova et al., 2015).

It is worth bearing in mind that emotional distress and unmet needs of the patients might vary significantly between different countries. It has been demonstrated that in comparison with their Western counterparts, Asian patients may develop more critical symptoms of emotional stress, which can negatively affect the caregivers' emotional health and quality of life (Lim et al., 2017).

Nipp et al. have reported that younger age, female gender, being married to the patient, and greater depression were significantly related to higher levels of anxiety among family caregivers of patients with cancer, which is in line with the findings of this study. They also have shown that nearly half of the family caregivers represented high rates of depression and anxiety (Nipp et al., 2016).

In this study, with regard to a cutoff score of 21, 83% of the caregivers were screened positive for burden, 30.7% of whom were in the severe category. Alsirafy et al. conducted a study on a population of family caregivers of incurable patients with cancer from Egypt and Saudi Arabia. In line with this study, they also assessed the caregiving burden using ZBI-22, and reported a 58.7% prevalence for significant caregiving burden (Alsirafy et al., 2021).

In a Nigerian study, only 4.4% of caregivers have found to be in a severe category, while most of the caregivers showed mild burden (44.5%). They have explained that a possible cause for such findings could be the embarrassment of caregivers to express their real burden due to their relationship with the patients (Onyeneho and Ilesanmi, 2021).

The positive correlation between depression and caregiving burden, as is proved in this study, has been demonstrated in a number of previous studies (Adelman et al., 2014; Seo and Park, 2019; Ahmad Zubaidi et al., 2020; Fang et al., 2022).

As discussed in a Malaysian study (Ahmad Zubaidi et al., 2020), among a population of informal caregivers in a palliative care unit, only half of the population were reported to experience caregiving burden, most of whom were in the mild-to-moderate burden category. Having symptoms of depression and anxiety along with being male, highly educated, caring for patients with cancer, and having long hours of caregiving were significant predictors of caregiving burden. In fact, caregivers with symptoms of depression and anxiety were 3 times more likely to bear the burden of caregiving. Conversely, caring for patients who do not have cancer has been associated with less likelihood of carrying the burden of caregiving. Such findings authenticate more challenges that caregivers may face in caring for patients with cancer compared to other chronic diseases.

In contrast to the aforementioned study, a review has indicated that female sex and low educational level are significant risk factors for caregiving burden, which is consistent with the finding of this study. Long durations of caregiving, depression, social isolation, financial stress, and lack of choice have also been proposed as other significant predicting factors of caregiving burden (Adelman et al., 2014). From the authors' point of view, the significant effect of educational level on the burden of caregiving could be explained in two ways. On the one hand, caregivers with higher levels of education may be properly informed of the prognosis of the disease and the challenges their patients would be facing through the cancer trajectory, which can result in an emotional burden on caregivers. On the other hand, caregivers with lower levels of education, to be specific, being illiterate or at the primary school level, may cause difficulties for the caregivers in terms of being actively involved in the process of cancer diagnosis and treatment and maintaining effective communication with healthcare centers and insurance companies.

In an Indian study, 70.22% of the cancer caregivers reported mild-to-moderate burden and 21.38% reported moderate-to-severe burden. They have indicated that the level of burden does not differ significantly according to marital status, education level, caregiver age group, and type of relationship to the patient even though they found gender (male) and employment status (unemployment) two significant factors associated with the high burden (Mishra et al., 2021).

In this study, the univariate analysis revealed some caregiver-related factors to be significantly associated with greater burden, including female sex, older age, lower educational level, being retired, and quitting for care, as well as longer durations of caregiving. These findings have profound implications for healthcare providers and for clinicians to take into consideration the influencing factors to manage the burden of cancer family caregivers.

In an Iranian population of cancer family caregivers, the prevalence of high caregiving burden was reported to be 48.1%. In the aforementioned study, four predicting variables including, being a spouse, caring for a male patient, being dissatisfied with family monthly income, and early cancer diagnosis (<1 month) have been suggested (Mirsoleymani et al., 2017). However, in this study, the gender of the patients was not significantly associated with the caregiving burden, caring for female patients was related to significantly more levels of anxiety.

A study has concluded that in comparison to more objective disease-related factors, e.g., stage of cancer, patient health-related quality of life seems to be more significantly influential on the burden perceived by family caregivers (Maguire et al., 2018). For instance, in terms of depression and anxiety, we showed that caregiving for patients who underwent surgery was related to significantly lower levels of these outcomes. One possible explanation for this observation might be linked to the patients' quality of life and psychological distresses which can have a significant impact on their caregivers' quality of life and psychological wellbeing. The findings of a recent systematic review support this explanation; it reveals that in a population of patients with small renal masses, undergoing active surveillance (AS) vs. surgery may be related to a significant reduction in total scores of Short Form-12 at enrollment and at the end of each follow-up period (2 and 3 years) (Vartolomei et al., 2022).

However, the existing evidence is in line with the fact that both disease-related and patient health-related quality of life factors might be crucial in predicting a higher burden among family caregivers (Thrush and Hyder, 2014; Mirsoleymani et al., 2017).

## Limitations

This study has some limitations. First, due to the cross-sectional nature of the study, further analysis to secure causal inferences was not possible. Second, we did not evaluate the validity of the instruments used to measure the outcome variables in this study. Third, this study was conducted using convenient sampling; therefore, this study cannot be generalized with its findings. Finally, we did not include participants who had been caring for their patients for periods of <6 months.

We strongly recommend conducting longitudinal studies and clinical trials considering a wide variety of participants in terms of cultural, economic, and social differences between countries.

## Conclusion

This study demonstrated a high prevalence of burden, anxiety, and depression among family caregivers of patients with cancer. Additionally, nine predicting factors for caregiving burden were found. Healthcare policymakers and clinicians should take these factors into consideration to take timely and effective measures, aimed at managing the burden and relieving psychological distress among cancer caregivers. Caregivers' wellbeing and welfare should be given close and thoughtful attention by healthcare providers. Neglecting caregivers' needs and burdens may create a disturbance to them in looking after their patients which for a long period of time could result in low quality of life and ill health among either patients or caregivers. From the authors' point of view, all related data to the main caregivers of patients with cancer must be documented to generate electronic data. Therefore, by means of these data, the caregivers of the patients can be periodically monitored by social workers and psychologists in terms of the caregiving burden, psychological consequences, and unmet needs.

## Data availability statement

The raw data supporting the conclusions of this article will be made available by the authors, without undue reservation.

## Ethics statement

The studies involving human participants were reviewed and approved by the Ethics Committee of Zanjan University of Medical Sciences [IR.ZUMS.REC.1398.105]. The patients/participants provided their written informed consent to participate in this study.

## Author contributions

ZK, MR, AZ, JM, OS, and SZ designed the study and provided the data and performed data analyses and quality control. ZK and AZ supervised the study. MR conducted the statistical analysis and drafted the manuscript and all authors contributed substantially to its revision. ZK takes responsibility for the paper as a whole. All authors read and approved the final manuscript.
